# ﻿*Neottiabifidus* (Orchidaceae, Epidendroideae, Neottieae), a new mycoheterotrophic species from Guizhou, China

**DOI:** 10.3897/phytokeys.229.103107

**Published:** 2023-07-28

**Authors:** Mei-Na Wang, Xin-Yi Wu, Cheng-Jiang Tan, Ping Yu, Wen-Hui Rao, Jie-Shan Chen, Jian Li, Jian-Bing Chen

**Affiliations:** 1 The Orchid Conservation & Research Center of Shenzhen and the National Orchid Conservation Center of China, Shenzhen Key Laboratory for Orchid Conservation and Utilization, Key Laboratory of National Forestry and Grassland Administration for Orchid Conservation and Utilization, Shenzhen, 518114, Guangdong, China Shenzhen Key Laboratory for Orchid Conservation and Utilization, Key Laboratory of National Forestry and Grassland Administration for Orchid Conservation and Utilization Shenzhen China; 2 Management Department of Maolan National Nature Reserve, Libo 558400, Guizhou, China Management Department of Maolan National Nature Reserve Libo China

**Keywords:** *Neottiabifidus*, new species, Orchidaceae, saprophytic orchid

## Abstract

*Neottiabifidus*, a new mycoheterotrophic orchid, found in Maolan National Nature Reserve in Guizhou Province, China, is described and illustrated here. The new species is close to *N.nidus-avis*, *N.kiusiana* and *N.papilligera* but differs in having a finely pubescent rachis with fewer flowers, a finely pubescent pedicel, and a fishtail-shaped lip that is deeply bilobed to the middle of the lip, with the lobes diverging at an acute angle (45°) to each other and mesochile with many papillae. Additionally, *N.bifidus* is well supported as a new species by molecular phylogenetic results based on ITS and chloroplast genome. The chloroplast genome of the novelty, which contains an LSC region of 33,819 bp, SSC region of 5,312 bp and IRs of 46,762 bp was assembled and annotated. A key to mycoheterotrophic *Neottia* species in China is also provided.

## ﻿Introduction

The genus *Neottia* Guett. comprises 81 accepted species, including 63 autotrophic species and 18 mycoheterotrophic species (https://powo.science.kew.org, Mu et al. 2017; Chen and Jin 2021), distributed widely in north temperate areas with a few species extending into alpine regions in the mountains of tropical Asia (Govaerts et al. 2019; Chen and Jin 2021). East Asia is one of the diversity centers for this genus with more than 70% of *Neottia* species occurring in this region (So and Lee 2020). Formerly, *Neottia* was divided into *Listera* and *Neottia* (Bentham 1881; Pfitzer 1887; Schlechter 1926; Brieger et al. 1974; Dressler 1981; Rasmussen 1982) by the distinct morphological differences possessed by autotrophic plants (*Listera*) with two opposite leaves (sometimes three or more) in the middle of the stem, while mycoheterotrophic plants are achlorophyllous and possess densely fleshy bird's nest like roots. In 2003, Govaerts cited another genus in Tribe Neottieae Lindl., *Holopogon*, as a synonym of *Neottia* (Govaerts 2003).

There are 52 species and one variation of *Neottia* in China, amongst which 14 species are mycoheterotrophic (https://powo.science.kew.org, Mu et al. 2017; Chen and Jin 2021). During our fieldwork in the Maolan National Nature Reserve, Libo County, Guizhou Province, China in 2021, an unknown species of mycoheterotrophic *Neottia* was found in the evergreen broad-leaved forest. Based on morphological characters and molecular evidence, it was considered as a new species of *Neottia* and is described below.

## ﻿Materials and methods

Morphological characteristics of the new species were observed, measured and photographed, based on living plants in Maolan National Nature Reserve, Guizhou. The studied specimens are deposited at The National Orchid Conservation Center of China and the Orchid Conservation & Research Center of Shenzhen. The general morphology was derived from fresh specimens and photographs were taken with a DSLR camera. To investigate the systematic position of the new species, the plastid genome and the nuclear ribosomal internal transcribed spacers (nrITS) marker were used in molecular phylogenetic analysis. Total genomic DNA was extracted from fresh flowers and stems (voucher specimens J.B.Chen 00599) using a plant genomic DNA kit and then sent to Novogene (Beijing, China) for the library (350 bp) preparation for genome skimming sequencing. Paired-end (150 bp) sequencing was conducted on the Illumina Hiseq 6000 platform (San Diego, CA), producing approximately 8 Gb reads. The plastid genome was assembled using GetOrganelle (Jin et al. 2020) with the chloroplast genome of *Neottiacamtschatea* (L.) Rchb. F.(NC_030707) and *Neottialisteroides* Lindl. (NC_030713) as the reference sequences. After assembly, the obtained scaffolds and contigs were annotated by Geneious Prime (Biomatters Ltd., Auckland, New Zealand) (Kearse et al. 2012) and Plastid Genome Annotator (Qu et al. 2019). The annotated complete chloroplast genome was deposited in GenBank with accession number OP279442. nrITS were also sequenced for the new species in this study. The PCR reactions and Sanger Sequencing were performed by Sangon Biotech (Shanghai, China). The primers used in this study are presented in Table 1. In total, 70 species (incl. 29 species of *Neottia*) from seven genera were used for molecular phylogenetic analyses (Table 2). The nrITS dataset consists of six genera and 66 species and the plastid genome dataset consists of five genera and 27 species, respectively. Five species of *Cionisaccus*, *Ophrys* and *Serapias* were selected as outgroup taxa based on Li et al. (2016). All plastid genomes were aligned by MAFFT 7.3 (ffT-NS-i × 1000 strategy) after removing one inverted repeat (IR) region of each sample (Katoh and Standley 2013). Poorly-aligned regions were removed by trimAl 1.2 with default settings before phylogenetic analyses (Capella-Gutiérrez et al. 2009). Maximum Likelihood (ML) analyses were conducted in IQTREE 1.6 using the SH-aLRT test and ultrafast bootstrap (UFBoot) feature (–alrt 1000 –bb 1000 –nt AUTO) (Nguyen et al. 2015; Hoang et al. 2018).

**Table 1.** Primers used in this study.

Primer	Sequence (5’to3’)	Origin
ITS-17SE	ACGAATTCATGGTCCGGTGAAGTGTTCG	Sun et al. 1994
ITS-26SE	TAGAATTCCCCGGTTCGCTCGCCGTTAC	Sun et al. 1994

**Table 2.** GenBank accession numbers for sequence data, a dash (-) indicates missing data and an asterisk (*) denotes sequences obtained in this study.

Species	nrITS	cp
*Aphyllorchiscaudata*	FJ454866	-
*Aphyllorchisgollanii*	MZ463253	-
*Aphyllorchismontana*	FJ454867	-
*Aphyllorchispallida*	MZ463252	-
*Cephalantherabijiangensis*	MZ463242	-
*Cephalantheradamasonium*	AY146446	NC_041179
*Cephalantheraepipactoides*	KY512499	-
*Cephalantheraerecta*	MZ463245	-
*Cephalantheraexigua*	FJ454868	-
*Cephalantherafalcata*	AB856493	-
Cephalantherafalcatavar.flava	MZ463241	-
*Cephalantherahumilis*	MZ463240	NC_030706
*Cephalantheralongibracteata*	MK306540	NC_041180
*Cephalantheralongifolia*	AY146447	NC_030704
*Cephalantherananchuanica*	JN706696	-
*Cephalantherananlingensis*	KT338669	-
*Cephalantherarubra*	AY146445	NC_041181
*Epipactisalbensis*	AY154384	NC_041182
*Epipactisatrorubens*	JN847403	-
*Epipactisduriensis*	AY351377	-
*Epipactisfageticola*	AY351382	-
*Epipactisflava*	FJ454869	-
*Epipactishelleborine*	MZ463247	MK608776
*Epipactisleptochila*	FJ454870	-
*Epipactislusitanica*	AY351381	-
*Epipactismairei*	MZ463250	NC_030705
*Epipactismicrophylla*	FR750399	MH590352
*Epipactismuelleri*	FJ454871	-
*Epipactispalustris*	AY146448	NC_041187
*Epipactispapillosa*	MZ463248	-
*Epipactispurpurata*	JN847416	MH590354
*Epipactisroyleana*	MZ463249	-
*Epipactisthunbergii*	MK306477	NC_046817
*Epipactisveratrifolia*	KF727435	NC_030708
*Epipactisvoethii*	FR750400	-
*Neottiaacuminata*	KT338755	-
*Neottiaalternifolia*	MZ463268	-
*Neottiabicallosa*	MZ463271	-
*Neottiabifidus*	OP265395*	OP279442*
*Neottiabifolia*	MG216639	-
*Neottiaborealis*	MG216431	-
*Neottiabrevicaulis*	MZ463258	-
*Neottiacamtschatea*	KJ023677	NC_030707
*Neottiacordata*	KJ023678	NC_041189
*Neottiasuzukii*	MH321188	NC_041447
*Neottiadivaricata*	MZ463257	-
*Neottiafugongensis*	MZ463256	NC_030711
*Neottiahybrid* sp.	MZ463255	-
*Neottiajaponica*	KT338756	NC_041446
*Neottiakaroana*	MZ463270	-
*Neottiakiusiana*	KT338757	MN537563
*Neottialisteroides*	MZ463262	NC_030713
*Neottiameifongensis*	MZ463267	-
*Neottiamucronata*	MZ463261	-
*Neottianidus-avis*	AY351383	JF325876
*Neottianujiangensis*	MZ463254	-
*Neottiaovata*	-	NC_030712
*Neottiapapilligera*	KT338758	-
*Neottiapinetorum*	KT338759	KU551269
*Neottiapuberula*	MH808061	-
*Neottiasmallii*	AF521058	-
*Neottiasmithiana*	MZ463263	-
*Neottiawardii*	MZ463260	-
*Neottiawuyishanensis*	MZ409849	-
*Cionisaccusprocera*	-	MW589517
*Ophrysapifera*	AY699976	-
*Ophrysfusca* subsp.	-	AP018716
*Ophrysinsectifera*	AY699950	-
*Ophryssphegodes*	-	AP018717
*Serapiascordigera*	AY364884	-

## ﻿Results

**Figure 1.**
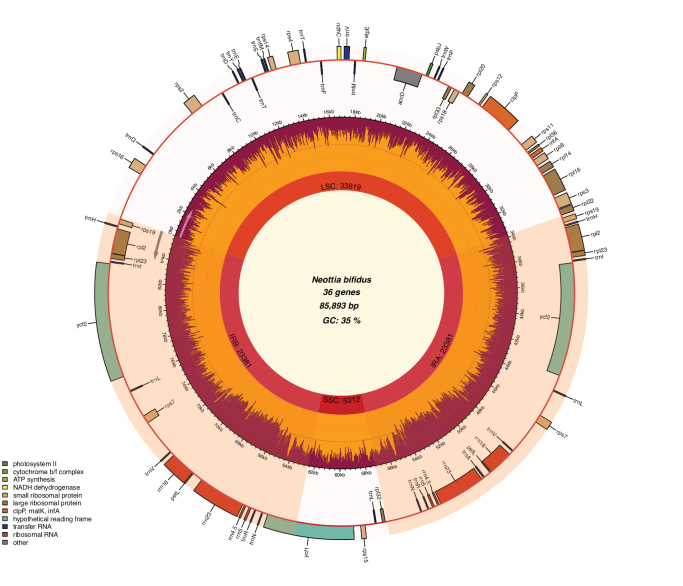
Chloroplast genome map of *N.bifidus*.

The whole chloroplast genome of *N.bifidus* showed a typical quadripartite structure containing a pair of inverted repeats (IRs) separated by a large single-copy (LSC) region and a small single-copy (SSC) region (Fig. 1). The complete plastid genome sequence of *N.bifidus* was 85,893 bp in length containing an LSC region of 33,819 bp, SSC region of 5,312 bp and IRs of 46,762 bp. The chloroplast genome contained 72 genes, including 36 protein-coding genes, 28 tRNA genes and eight rRNA genes (Table 3). The overall GC content is 35%.

**Table 3.** Genes present in the chloroplast genome of *Neottiabifidus*.

Group of genes	Gene
Photosystem I	-
Photosystem II	*psbJ*
Cytochrome b/f complex	petL*
ATP synthase	*atpE*
NADH dehydrogenase	*ndhC*
Rubis CO large subunit gene	-
RNA polymerase	-
Small ribosomal proteins	rps2, rps3, rps4, rps7*, rps8, rps11, rps12, rps14, rps15, rps16, rps18, rps19*
Large ribosomal proteins	rpl2*, rpl14, rpl16, rpl20, rpl22, rpl23*, rpl32, rpl33, rpl36
tRNA	trnA-UGC, trnC-GCA, trnD-GUC, trnE-UUC, trnF-GAA, trnfM-CAU, trnH-GUG*, trnI-CAU*, trnL-CAA*, trnL-UAG, trnM-CAU, trnN-GUU*, trnP-UGG, trnQ-UUG, trnR-ACG*, trnS-UGA, trnT-GGU, trnT-UGU, trnV-GAC*, trnV-UAC, trnW-CCA, trnY-GUA
rRNA	rrn4.5*, rrn5*, rrn16*, rrn23*
Translational initiation factor	*infA*
Subunits of Acetyl-CoA-carboxylase	*accD*
Protease	*clpP*
Conserved open reading frames	ycf1, ycf2*

Note: * means duplicated gene in IRs.

**Figure 2.**
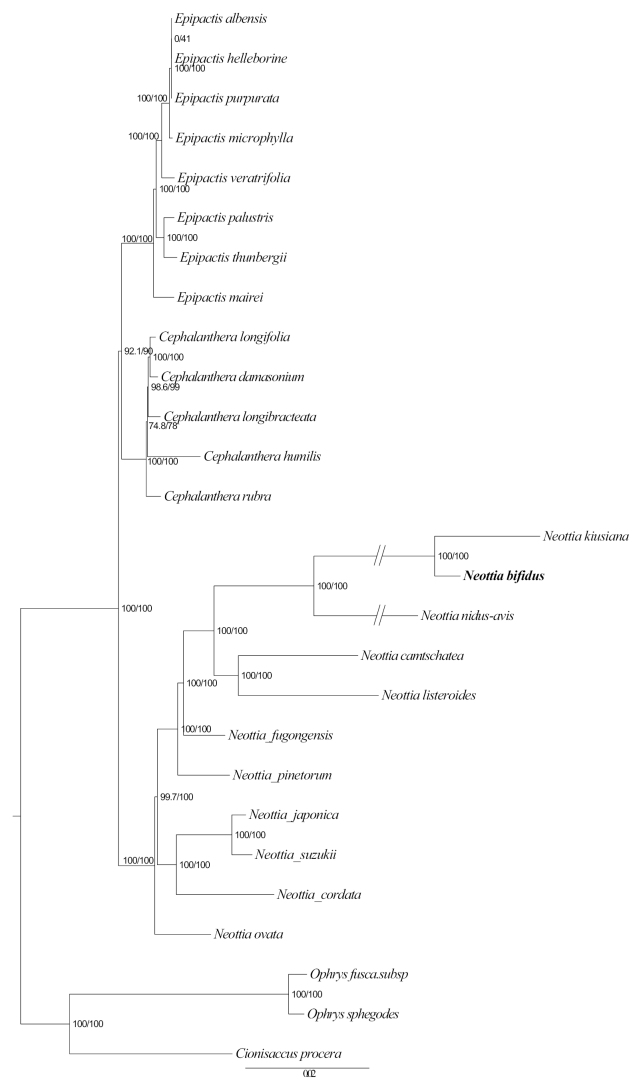
Phylogram of *Neottieae*, based on the plastid genome. The numbers near the nodes are the values of SH-aLRT test (left) and the ultrafast bootstrap (right).

**Figure 3.**
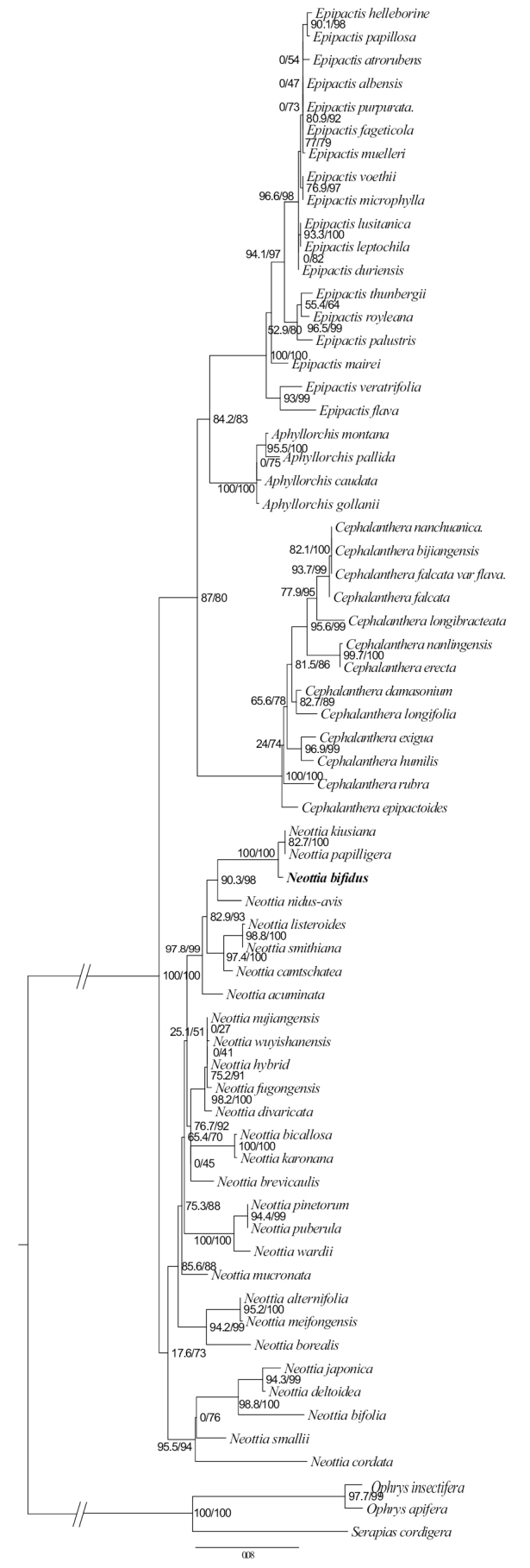
Phylogram of Neottieae, based on nrITS. The numbers near the nodes are the values of SH-aLRT test (left) and the ultrafast bootstraps (right).

The phylogenetic analyses indicated that this unknown species is far from other autotrophic species, but has a better clustering relationship with leafless holomycotrophic species in *Neottia*. The phylogenetic tree, based on the plastid genome, indicated that it is close to *N.kiusiana* T.Hashim. & S.Hatus. (KT338757) with high support (SH-aLRT 100%, UfBoot 100%) and then sister to *N.nidus-avis* (L.) Rich. (JF325876) also with strong support (SH-aLRT 100%, UfBoot 100%) (Fig. 2). The phylogenetic tree, based on nrITS, showed that the new species is sister to *N.kiusiana* and *N.papilligera* Schltr. with high support (SH-aLRT 100%, UfBoot 100%) (Fig. 3).

### ﻿Taxonomy

#### ﻿ Neottia bifidus

Taxon classificationPlantaeAsparagalesOrchidaceae

M.N.Wang sp. nov.

0410D15E-A8CF-5619-96E6-8BE5ED9F437F

urn:lsid:ipni.org:names:77324361-1

**Figure 4.**
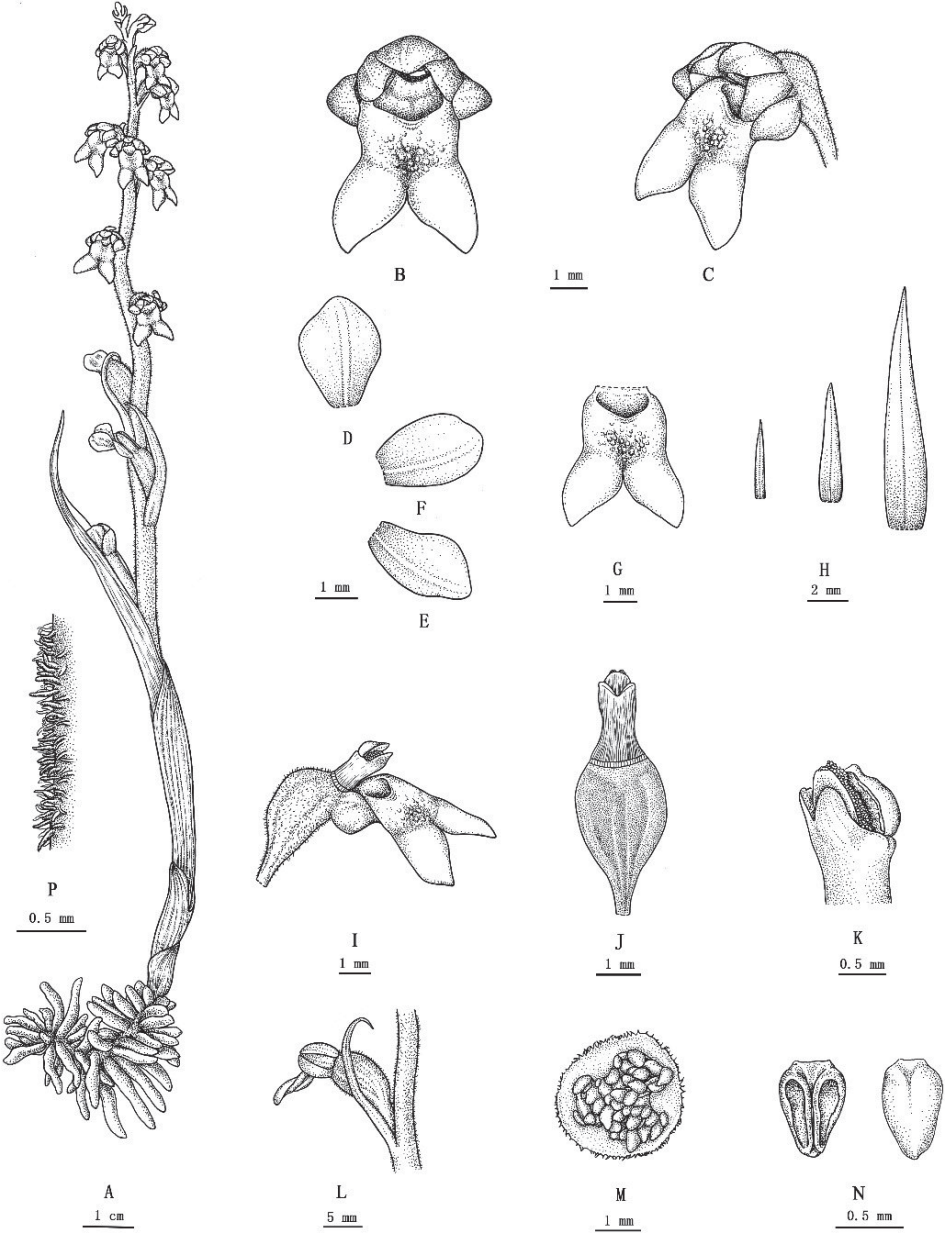
*Neottiabifidus* M.N.Wang, sp. nov. **A** whole plant **B** flower (front view) **C** flower (side view) **D** dorsal sepal **E** lateral sepal **F** petal **G** lip (front view) **H** bracts **I** ovary, column and lip (side view) **J** ovary and column (ventral view) **K** column **L** fruit with bract **M** fruit (cross section) **N** anther cap **P** hairy on rachis.

**Figure 5.**
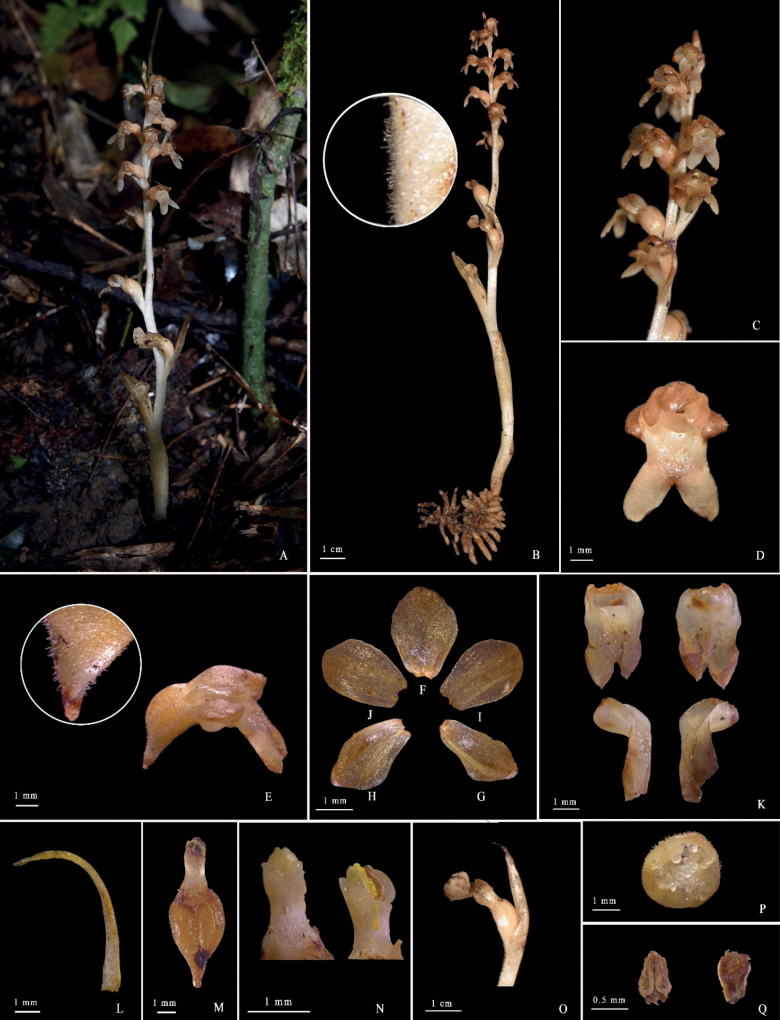
*Neottiabifidus* M.N.Wang, sp. nov. Photographed by M. N. Wang & W. H. Rao. **A** habit **B** whole plant and hairy on rachis **C** inflorescence **D** flower (front view) **E** ovary and flower (side view) and hairy on ovary **F** dorsal sepal **G, H** lateral sepals **I, J** petals **K** lip (front view, back view and side view) **L** bract **M** ovary and column **N** column **O** fruit with bract **P** fruit (cross section) **Q** Anther cap.

Figs 4 , 5 Chinese name: 鱼尾鸟巢兰

##### Type.

China. Guizhou Province, Qiannan Buyi and Miao Autonomous Prefecture, Libo County, the Maolan National Nature Reserve, 825 m elev., 23 April 2021, J.B.Chen 00599 (holotype: NOCC).

##### Diagnosis.

*Neottiabifidus* is morphologically similar to *N.nidus-avis*, *N.kiusiana* and *N.papilligera* but differs in having a finely pubescent rachis, with fewer flowers; finely pubescent pedicel; and fish-tail-shaped lip, deeply 2-lobed to the centre of mid-lip, lobes diverging at an acute angle (45°) to one another, mesochile with many papilloses (Table 4).

**Table 4.** Morphological comparison of *Neottiabifidus* and similar species.

Morphological characters	*N.bifidus*	*N.kiusiana* (Yukawa et al. 2009)	*N.papilligera* (Chen et al. 2009)	*N.nidus-avis* (Jersáková et al. 2022)
Plant height	15–19 cm	6–21 cm	27–30 cm	15–60 cm
Rachis	Rachis densely pubscent, laxly and irregularly 9–15-flowered.	Rachis sparsely glandular hairy, with 10–28 flowers.	Rachis glabrous or pubescent, with much more than 20 flowers.	Rachis glabrous, with much more than 20 flowers.
Pedicel	Pubscent	Glabrous	Glabrous	Glabrous
Lip	Lip 2-lobed to the centre of mid-lip; hypochile without purple dots; mesochile with many papilloses; epichile 2-lobed, lobes triangular, fish-tail-shaped, diverging at an acute angle (45°) to one another.	Lip 2-lobed (not up to the centre of mid-lip); hypochile purple-dotted adaxially; epichile 2-lobed, lobes transversely oblique-rectangular, rectangular or oblong, diverging at an acute angle (45°) to one another.	Lip apex deeply 2-lobed; lobes narrowly oblong, usually twisted, diverging at an obtuse angle (120°–170°) to one another.	Lip apex deeply 2-lobed, diverging at an obtuse angle (120°–170°) to one another.

Terrestrial herbs, leafless, holomycotrophic, 10–19 cm tall. Rhizome short, with many stout, fleshy fascicled roots. Stem erect, terete, leafless, pubscent, with 2–3 sheaths at base; sheaths tubular, 2–3 cm, membranous, glabrous, with 4–7 dark brown veins, upper ones much longer than lower ones; rachis 7–13 cm, pubscent, laxly and irregularly 9–15-flowered; floral bracts membranous, glabrous, narrowly lanceolate, ovate-lanceolate, obtuse to subacute, 0.7–2.1 cm long, lowermost ones much longer than flowers, 1.1–1.3 × 2.6–3 cm, gradually diminishing in upper ones which are shorter than ovaries. Flowers resupinate, pale brown; pedicel and ovary 0.6–1.5 cm long, pubescent. Sepals membranous, ovate to obovate, pale brown, nearly equal in size; dorsal sepal cucullate, 2.3–2.4 × 1.6–1.8 mm, apex obtuse, glabrous; lateral sepals cucullate, strongly cupped, 2.4–2.5 × 1.4–1.5 mm, apex obtuse, glabrous. Petals membranous, ovate to obovate, pale brown, nearly equal in size to dorsal sepal. Lip spreading downwards, subrectangular, 3.8–5 mm long, small and semi-transparent at early anthesis, becoming larger and yellowish-brown at late anthesis, apex deeply 2-lobed to the center of mid-lip; hypochile rectangular, concave at base; mesochile with many papilloses; epichile 2-lobed, lobes extending outwards, triangular, fish-tail-shaped, 2.3–2.5 × 1.5–1.6 mm, diverging at an acute angle (45°) to one another, apex obtuse, margins of apices and inner sides repand or erose. Column cylindrical, 2.8–3 mm long; anther inclined towards rostellum, elliptic, ca. 0.7 mm; stigma ca. 0.9 mm, lamellate, 2-lobed; rostellum shorter than anther. Capsule elliptic, with persistent sepals and petals, 1–1.5 cm long.

##### Etymology.

The species epithet refers to the fish-tail-shaped lip of the new species.

##### Distribution and habitat.

*Neottiabifidus* is currently known only from the type locality in Libo, Guizhou, China. It grows in humus-rich soil under broad-leaved forests at elevations of 700–900 m and is found growing with *Miliusasinensis* Finet & Gagnep. (Annonaceae), *Platycaryastrobilacea* Siebold & Zucc (Juglandaceae), *Micheliamartini* (H. Lév.) Finet & Gagnep. ex H. Lév. (Magnoliaceae), *Mallotusphilippensis* (Lamarck) Müll. Arg. (Euphorbiaceae), *Symplocosadenophylla* Wall. (Symplocaceae), *Chimonobambusaangustifolia* C. D. Chu & C. S. Chao (Poaceae), *Murrayaexotica* L. (Rutaceae), *Gomphandratetrandra* (Wall.) Sleumer (Stemonuraceae), *Diospyrosmollis* Griff. (Ebenaceae), *Strobilantheshongii* Y. F. Deng & F. L. Chen (Acanthaceae), etc.

##### Phenology.

Flowering and fruiting from Apr–May.

##### Conservation status.

During our fieldwork, only one population with less than 10 individuals was discovered in Maolan National Nature Reserves (213 km^2^). Most individuals were found growing along the roadside and are easily disturbed by human activities. According to the guidelines for using the IUCN Red List Categories and Criteria (IUCN 2022), the new species should be temporarily assigned as ‘Critically Endangered’ by its limited populations, localities and vulnerable habitats.

##### Note.

*Neottiabifidus* is morphologically - related to three species, namely, *N.nidus-avis*, *N.kiusiana* and *N.papilligera*, but it is readily distinguished from them, based on morphological characters given in Table 4.

### ﻿Key to mycoheterotrophic species of *Neottia* in China

**Table:** 

1	Stigma terminal; rostellum absent	**2**
–	Stigma lateral or rarely subterminal; rostellum present, usually above concave stigma	**4**
2	Flowers purplish-red	***Neottiagaudissartii* (*Holopogongaudissartii*)**
–	Flowers green	**3**
3	Flowers actinomorphic, lip very similar to the petals	***N.pekinensis* (*Holopogonpekinensis*)**
–	Flowers zygomorphic, lip bilobed at the apex, utterly different from the petals	***N.smithiana* (*Holopogonsmithianus*)**
4	Lip entire; column (excluding anther and rostellum) less than 0.5 mm	**5**
–	Lip bilobed at apex; column (excluding anther and rostellum) 1.5–4 mm	**6**
5	Floral rachis glabrous; flowers resupinate	***N.acuminata***
–	Floral rachis villous; flowers not resupinate	***N.taibaishanensis***
6	Lip distinctly concave at base	**7**
–	Lip not concave at base	**9**
7	Apical lobes of lip parallel or diverging at an acute angle to one another	***N.bifidus***
–	Apical lobes of lip diverging at an obtuse angle to one another	**8**
8	Apical lobes of lip 2.5–3 mm; sinus of lip without a short tooth between lobes	***N.papilligera***
–	Apical lobes of lip less than 1 mm; sinus of lip with a short tooth between lobes	***N.brevilabris***
9	Lip with a pair of triangular auricles at base	***N.tenii***
–	Lip without a pair of auricles at base	**10**
10	Lip obovate, 6–10 mm wide	***N.megalochila***
–	Lip narrowly obovate-oblong or cuneate, 1.5–4 mm wide	**11**
11	Lip narrowly obovate-oblong, 6–9 × 3–4 mm	***N.listeroides***
–	Lip cuneate, 10–12 × 1.5–2 mm	***N.camtschatea***

## Supplementary Material

XML Treatment for Neottia bifidus

## ﻿Additional information

### Author ORCIDs

Mei-Na Wang https://orcid.org/0000-0001-5998-6028

Xin-Yi Wu https://orcid.org/0000-0002-8623-1867

Cheng-Jiang Tan https://orcid.org/0009-0008-4558-4907

Ping Yu https://orcid.org/0009-0003-3319-7145

Wen-Hui Rao https://orcid.org/0000-0002-2177-6700

Jie-Shan Chen https://orcid.org/0009-0000-8859-6053

Jian Li https://orcid.org/0000-0002-0096-6257

Jian-Bing Chen https://orcid.org/0000-0001-9085-0663

### Data availability

All of the data that support the findings of this study are available in the main text.
